# Effect of a bacterial glutaminyl cyclase inhibitor on multi-species-biofilms

**DOI:** 10.3389/froh.2025.1716625

**Published:** 2025-12-01

**Authors:** Sigrun Eick, Nadine Taudte, Daniel Ramsbeck, Anna Magdoń, Anton Sculean, Jan Potempa, Mirko Buchholz

**Affiliations:** 1 Department of Periodontology, School of Dental Medicine, University of Bern, Bern, Switzerland; 2 Perio Trap Pharmaceutical GmbH, Halle/Saale, Germany; 3 Department of Molecular Drug Design and Target Validation, Fraunhofer Institute for Cell Therapy and Immunology, Halle (Saale), Germany; 4 Department of Microbiology, Faculty of Biochemistry Biophysics and Biotechnology, Jagiellonian University, Kraków, Poland; 5 Department of Oral Immunology and Infectious Diseases, School of Dentistry, University of Louisville, Louisville, KY, United States

**Keywords:** modifying bacterial virulence, *Porphyromonas gingivalis*, alternatives antibiotics, biofilm modulation, periodontitis

## Abstract

**Introduction:**

Modifying bacterial virulence could be an interesting alternative to antibiotics. The study aimed to examine the effects of an inhibitor targeting bacterial glutaminyl cyclase [which is selectively present in *Porphyromonas gingivalis* (*Pg*), *Tannerella forsythia* (*Tf*), and *Prevotella intermedia* (*Pi*)] on various multispecies biofilms.

**Methods:**

Two multi-species biofilms—one containing four species (including *Tf)* and another with 12 species (including *Tf*, *Pg*, and *Pi*)—were cultured in the presence of 31.25–500 µM of a [4,5-c]pyridine-based inhibitor. After 24 h, bacterial counts, biofilm biomass, metabolic activity, and, when *Pg* was included, Arg-gingipain activity were measured. Additionally, the biofilms were exposed to monocytic cells; here, the release of interleukin (IL)-1β and IL-10 was analyzed. The data were analyzed using a one-way analysis of variance (ANOVA) with a *post-hoc* comparison performed using the Bonferroni correction.

**Results and Discussion:**

In all biofilms, total bacterial counts and those of *Pg* and *Tf* remained unaffected by the inhibitor. In the 12-species biofilm, both biomass and total metabolic activity decreased at high inhibitor concentrations (500 µM to 75.2 ± 6.5% and 87.2 ± 5.8%, respectively; each *p* < 0.001). The arginine-specific amidolytic activities of Rgp declined dose-dependently, down to 60.4 ± 10.2% (*p* < 0.001) at 500 µM of the inhibitor. Consequently, *Pg* colonies lost pigmentation as inhibitor concentrations increased. The inhibitor also reduced IL-1β release from monocytic cells stimulated by the 12-species biofilm. The studied [4,5-c]pyridine-based inhibitor is able to modify virulence of a multispecies biofilm. It might have the potential to be a promising approach in periodontal prevention and therapy.

## 1 Introduction

Periodontitis is a chronic disease that affects the tissues surrounding the teeth and can lead to tooth loss if left untreated. It is one of the most prevalent diseases worldwide; a recent systematic review estimated a prevalence of 61.6% among adult dentate individuals (1). The disease is triggered by a dysbiotic microbiota, which causes an increase in subtypes of epithelial cells and fibroblasts with an inflammatory signature, a dysregulated neutrophil response, an increase in pro-inflammatory (M1) macrophages, and lymphocytes with innate immune properties. All these factors together activate NF-κB signaling, which promotes bone resorption and suppresses the expression of bone matrix proteins (48). The transformation of the eubiotic microbiota into a dysbiotic state associated with periodontal disease has been linked to *Porphyromonas gingivalis*. This keystone pathogen can impair the host's protective response while promoting inflammation (2). Major virulence factors of *P. gingivalis* include its two arginine-specific (RgpA, RgpB) and one lysine-specific (Kgp) cysteine proteases, also known as gingipains (3). Other bacterial species, such as *Tannerella forsythia* and *Treponema denticola*, are designated as inflammatory pathobionts, as their virulence genes are upregulated only when tissue destruction has already occurred (2). The subgingival microbiota consists of hundreds of species. Among those enriched in periodontitis compared to periodontal health are, in addition to the previously mentioned three species, *Prevotella intermedia*, various *Fusobacterium nucleatum* subspecies, *Filifactor alocis*, *Fretibacterium* species, and *Saccharibacteria* TM7 (4).

According to the European Federation of Periodontology (EFP) S3 level clinical practice guideline (5), the treatment of periodontal disease involves several steps. The first step focuses on controlling supragingival biofilm, gingival inflammation, and risk factors. The second step includes instrumentation, with or without adjuncts. Patients who do not respond adequately receive additional treatment, such as periodontal surgery, in the third step. Finally, all patients should participate in a supportive care program at intervals of 3–12 months. As before, controlling supragingival biofilm, gingival inflammation, and risk factors remain essential parts of this stage of therapy (5).

As the dysbiotic biofilm plays a crucial role in disease development, the use of antiseptics and/or antibiotics is discussed in periodontal treatment. According to the guidelines mentioned earlier, the adjunctive use of antiseptics (chlorhexidine) may be considered in the second step of therapy (5). Chlorhexidine is a bisbiguanide and is widely used in dental practice (6). It acts quickly to kill many bacteria (7). Specifically, when included in a mouthwash, it effectively prevents new biofilm formation (8). However, adverse effects like tooth discoloration, taste changes, irritation of the oral mucosa or tongue are commonly reported (8, 9).

Using systemic antibiotics as an adjunct improves clinical outcomes (10). However, routine use is not advised; it should only be considered for severe periodontitis in young adults (5). A recent analysis supports this, showing that a combination of amoxicillin and metronidazole effectively halts disease progression, especially in patients with generalized periodontitis at stage III/grade C (11). Antibiotics can cause side effects like gastrointestinal issues, metallic taste, and nausea (10). Additionally, the rise of antimicrobial resistance has become a growing global concern, with nearly 5 million deaths estimated in 2019 linked to infections caused by antibiotic-resistant bacteria (12). One of the key factors contributing to this increase is the widespread use of antibiotics (13).

This suggests that antibiotic use should be limited and alternative options explored. Altering bacterial virulence factors, such as gingipains, presents an interesting approach (14). One potential target could be glutaminyl cyclase (QC) in *P. gingivalis*. This enzyme is a mammalian-like type II enzyme that catalyzes the cyclization of glutamine (Q) to pyroglutamate (15, 16). Most proteins secreted by *P. gingivalis* via the type 9 secretion system (T9SS), including gingipains, and those exported into the periplasm are transported through the inner membrane via the Sec pathway, where the signal peptide is cleaved by a signal peptidase (17). Downstream of this cleavage site, the proteins often carry a Q, which seems to be cyclized by QC attached to the inner membrane on the periplasmic side (15). Besides *P. gingivalis*, a QC is also found in *T. forsythia* and *P. intermedia* (15, 16). The first tested QC inhibitor was able to suppress growth in these three species but did not impact other oral bacteria (16). Further development led to the discovery of a new class of *P. gingivalis* QC inhibitors based on a tetrahydroimidazo[4,5-c]pyridine scaffold (18). Selected compounds could inhibit the QC of *P. gingivalis* and *P. intermedia* at concentrations around 0.1 µM, and those of *T. forsythia* at 0.2 µM (18).

The purpose of the experiments outlined below was to determine the effects of a newly developed [4,5-c]pyridine-based QC inhibitor (S-0636) on two different multispecies biofilms. It was shown that the inhibitor does not affect viability of *P. gingivalis*, that it inhibits hemagglutination and invasion of *P. gingivalis* into keratinocytes and that it is potentially safe to host cells (Taudte et al. manuscript sumitted (19)). The key questions were: a) Does the inhibitor impact the biofilm overall structure, including biofilm biomass covering bacteria and matrix, total bacterial counts, and metabolic activity? b) Does it influence specific bacterial populations (such as commensals and pathobionts like *T. forsythia*, *P. gingivalis, P. intermedia*)? c) Does it affect bacterial virulence, specifically the Arg-gingipain activity of *P. gingivalis*? d) What concentration of the inhibitor disrupts the biofilm? e) Are there differences between the different types of biofilms (4-species without *P. gingivalis*, and 12-species)? and f) Does pretreating the biofilm with the inhibitor modulate the immune response?

## 2 Materials and methods

### 2.1 Inhibitors

From a panel of different compounds, a [4,5-c]pyridine-based inhibitor was selected: S-0636. Its synthesis is described elsewhere (19). It was dissolved in phosphate buffered saline (PBS, Gibco™ PBS pH 7.4,Thermofisher Scientific, Waltham, MA, USA). The inhibitor was used in at final concentrations of 500 µM, 250 µM, 125 µM, 62.5 µM, 31.25 µM in the assays. PBS served as a negative control.

### 2.2 Microorganisms in defined biofilm

Two different biofilms were used. The four-species biofilm included *Streptococcus gordonii* ATCC 10558*, Actinomyces naeslundii* ATCC 12104, *Fusobacterium nucleatum* ATCC 25586, and *Tannerella forsythia* ATCC 43037. For the 12-species biofilm, the following species were additionally included: *Porphyromonas gingivalis* ATCC 33277, *Prevotella intermedia* ATCC 25611, *Campylobacter rectus* ATCC 33238, *Capnocytophaga gingivalis* ATCC 33624, *Eikenella corrodens* ATCC 23834, *Filifactor alocis* ATCC 33238, *Parvimonas micra* ATCC 33270, and *Treponema denticola* ATCC 35405.

The strains were passaged on tryptic-soy agar plates (Oxoid, Basingstoke, GB) with 5% sheep blood (and with 10 mg/L N-acetylic muramic acid for *T. forsythia*). *T. denticola* ATCC 35405 was maintained in modified mycoplasma broth (BD, Franklin Lake, NJ) enriched with 1 g/mL cysteine, and 5 μg/mL cocarboxylase. All the strains were cultured at 37 °C, streptococci, and *A. naeslundii* ATCC 12104 in 10% of CO_2_, the other strains under anaerobic conditions.

### 2.3 Biofilm formation

The biofilm formation followed our recently published protocols (20, 21). Ninety-six-well plates were coated with 10 µL/well of a protein solution containing 0.67% mucin (Merck KGaA, Darmstadt, Germany) and 1.5% (w/v) bovine serum albumin (SERVA Electrophoresis GmbH, Heidelberg, Germany) for 15 min.

Twenty-four hours cultures of bacterial strains were scraped from agar-plates suspended in 0.9% w/v NaCl solution based on OD600nm = 1 (about 1.2 × 10^9^ bacteria/mL). Then, the bacterial mixture was prepared using 1 part *S. gordonii*, 2 parts *A. naeslundii* and 4 parts of the other strains except *T. forsythia*, *P. gingivalis* and *T. denticola* (in case of the 4-species biofilm 1 part *S. gordonii*, 2 parts *A. naeslundii*, each 4 parts *F. nucleatum*, *T. forsythia*)*.* This mixture was added in a ratio 1: 9 to the culture medium [Wilkins-Chalgren broth (Oxoid, Basingstoke, GB)] with 10 mg/L β-NAD + 10 mg/L N-acetylic muramic acid. From this suspension, 200 µL was pipetted per well and the plate was incubated for 6 h under anaerobic conditions. Afterward, 50 µL of a suspension (prepared as before for the other strains) containing *T. forsythia* (*P. gingivalis* and *T. denticola* according to the experiments) along with the inhibitor at the respective concentration or a control, was added (final volume 250 µL). Following an additional 18 h of incubation (total 24 h) in anaerobic conditions, the samples were analyzed.

### 2.4 Analysis of biofilms

The supernatant of the biofilm was removed and the biofilms were briefly and carefully washed once. Then, 250 µL of 0.9% w/v NaCl was added. The biofilms were scraped from the surface and thoroughly mixed. From that suspension, 25 µL were used to determine colony forming unit counts (CFU), 25 µL for measuring metabolic activity, 25 µL for biofilm biomass determination, 25 µL for qPCR, and 100 µL for quantifying the arginine-specific amidolylic activity (BApNA).

A 10-fold dilution series was performed on the aliquot designated for CFU determination. Each 25 µL sample was plated on tryptic-soy-agar plates (Oxoid) supplemented with 5% sheep blood. One series was incubated under anerobic conditions (representing total CFU counts), while the other was incubated with 10% CO_2_ to count of streptococci and actinomyces (as commensals)). Measurement of metabolic activity and of biofilm omass followed recently published methods (22–24). For measuring metabolic activity, 200 mg/L resazurine (Merck KGaA) was mixed with nutrient media at a ratio 1:10, each 100 µL per well was added to the biofilm suspension in a 96-well-plate. After 1-hour incubation at 37  °C, the plate was read at 570 nm against 600 nm. For quantification the biofilm biomass, biofilm aliquots were pipetted into a new 96-well-plate and fixed on the surface at 60  °C for 60 min. Next, 50 µL of a 0.06% (w/v) crystal violet solution was added per well. After washing, 50 µL of 30% acetic acid were added, and after 10 min the plate was read at 600 nm.

The counts of *T. forsythia*, *P. gingivalis* and *P. intermedia* were determined by using qPCR (25). Additionally, in a 12-species biofilm (including *P. gingivalis*), the arginine-specific amidolytic activity was measured. The respective suspension was centrifuged for 10 min with 5,000 g at 20  °C. To the sediment, 200 µL NaCl was added, the mixture was exposed to ultrasonication to breakdown the cell wall of bacteria, ensuring that cell-wall activity was also included the measurements. The amidolytic activity was monitored at 405 nm for 1 h at 37  °C after adding 0.5 mM N-a-benzoyl-DLarginine-p-nitroanilide (BApNA; Merck KgaA) in 1.0 mL of 0.2 M Tris–HCl, 0.1 M NaCl, 5 mM CaCl_2_, 10 mM cysteine, pH 7.6.

Additionally, in selected samples (12-species biofilm, 500 µM of inhibitor and control), the expression of gingipains was quantified and the location of *P. gingivalis* and *T. forsythia* was visualized. To quantify gingipain expression, RNA was extracted, cDNA was synthesized, and qPCR was performed as described previously (26).

For visualization by FISH technology, biofilms were cultured as before but on glass slides in 24-well plates. Samples have been were fixed in 4% paraformaldehyde/PBS for 1 h. After washing in PBS and preincubating in hybridization buffer (20% formamide, 0.9 M NaCl, 20 mM Tris-HCl, 0.01% SDS) at 46  °C for 15 min, the probes for *P. gingivalis* [Pg-Cy3 (27)], *T. forsythia* (Tf-Atto-488, sequence (27) and all bacteria [EUB 338-Atto-425, sequence (28)] in concentrations of 1 µM, 1.5 µM and 0.5 µM were added to the hybridization buffer and incubated for 3 h at 46  °C. After several washing steps, samples were embedded and visualized using a Zeiss LSM 710 confocal microscope (Carl Zeiss). Additional visualization was conducted with *Imarisviewer* software (Bitplane, *IMARIS* 10.0.0).

### 2.5 Interaction of monocytic cells with biofilm

The biofilms were formed as described above for 24 h.

MONO-MAC-6-cells (DSMZ no. ACC 124), a monocytic cell line of human origin, were cultured with RPMI 1640 medium (Invitrogen; ThermoFisher Scientific, Waltham, MA, USA) supplemented with 10% fetale bovine serum (FBS; Invitrogen). Before use in the experiments, MONO-MAC-6 cells were washed once and adjusted to 10^5^ cells/mL in RPMI 1640 medium with 0.5% v/v FBS and the inhibitor at the respective concentration.

The supernatants of the biofilms were carefully removed and replaced with 200 µL/well of MONO-MAC-6 cell suspension, with and without inhibitor. There is always also a cell control w/o biofilm. After 4 h of incubation, the media were taken out, centrifuged at 250 *g* at 20  °C for 10 min. The supernatants were stored at −80  °C until analysis. Commercially ELISA kits (R&D Systems, Minnesota, MN, USA) were used to measure the levels of released interleukin IL-1β and IL-10 according to the manufacturer's instructions. The detection limit was 1 pg/mL for both cytokines.

An influence on cell vitality by the experimental settings was screened using the MTT assay (29).

### 2.6 Statistical analysis

In all biofilm experiments, each of eight independent biological samples (obtained in two series) was included in the statistical analysis. Bacterial counts were recorded as log10. In presentations, the arbitrary units of activity and the mass data of the biofilm were related to the means of the untreated controls. The data were compared using a one-way analysis of variance (ANOVA) with a *post-hoc* comparison using Bonferroni correction. The statistical analysis was performed with SPSS 29.0 (IBM, Armonk, NY, USA).

## 3 Results

Of the *post-hoc* analyses, only statistically significant differences vs. control are presented.

### 3.1 Four-species biofilm

**Figure 1:**
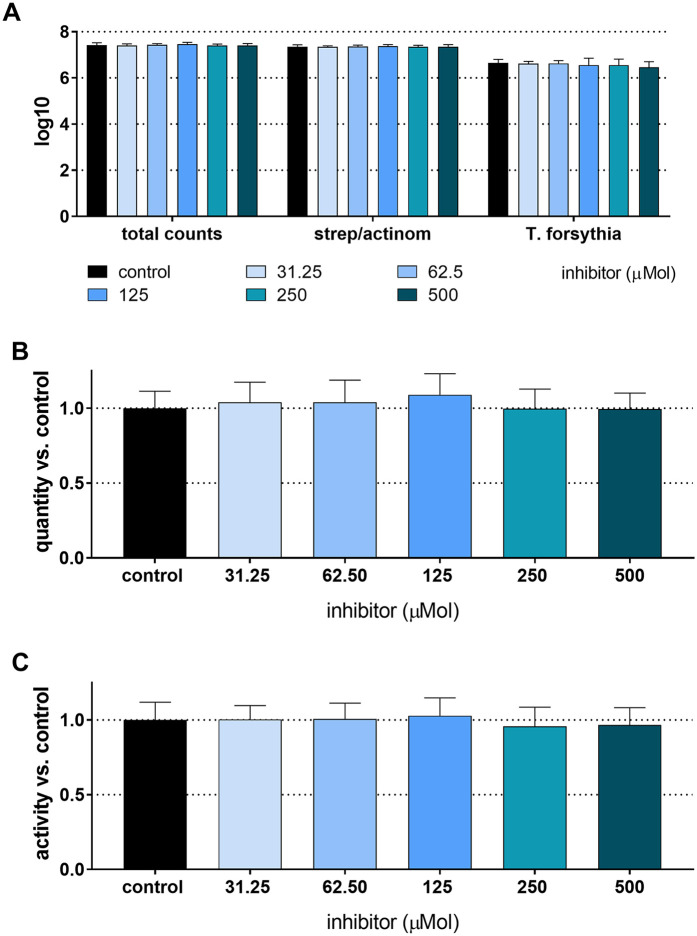
Total counts and counts of streptococci/actinomyces and *Tannerella forsythia* **(A)**, biomass **(B)**, and metabolic activity **(C)** of 24 h 4-species biofilms cultured with and without different concentrations of S-0636.

The total CFU counts of the 4-species biofilm without the addition of an inhibitor were 7.42 ± 0.10 log10, and the counts of *T. forsythia* were 6.65 ± 0.15 log10. There was no statistically significant difference in bacterial counts, biofilm activity, or biofilm biomass when any concentration of the inhibitor was added (Figure 1).

### 3.2 Twelve-species biofilm

**Figure 2:**
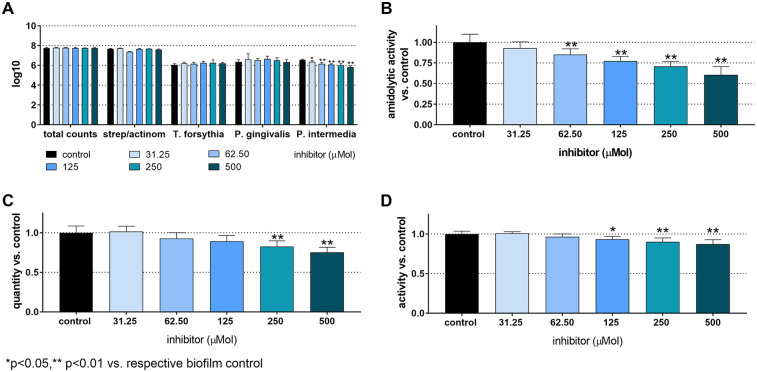
Total counts and counts of streptococci/actinomyces, *Tannerella forsythia*, *Porphyromonas gingivalis*, and *Prevotella intermedia* **(A)**, arginine-specific amidolytic activity **(B)**, biomass **(C)**, and metabolic activity **(D)** of 24 h 12-species biofilms cultured without and with different concentrations of S-0636. *p < 0.05, **p < 0.01 vs. control.

The total CFU counts in the 12-species biofilm without added inhibitor were 7.75 ± 0.05 log10. *T. forsythia* counts were 6.05 ± 0.12 log10, *P. gingivalis* counts were 6.37 ± 0.23 log10, and *P. intermedia* counts were 6.55 ± 0.09 log10. No bacterial counts showed a statistically significant difference, except for *P. intermedia*, which decreased in a concentration-dependent manner. The lowest counts occured at 500 µM of the inhibitor (5.84 ± 0.11 log10). The biofilm biomass was statistically significantly reduced, when treated with 250 µM (82.7 ± 7%, *p* < 0.001) and 500 µM of the inhibitor (75.2 ± 6.5%, *p* < 0.001). Additionally, the total biofilm metabolic activity decreased statistically significantly at inhibitor concentrations of 125 µM and higher (125 µM to 93.3 ± 3.7%, *p* = 0.033; 250 µM to 90.3 ± 4.8%, *p* < 0.001; 500 µM to 87.2 ± 5.8%, *p* < 0.001). The arginine-specific amidolytic activities of the biofilm decreased in a concentration-dependent manner with the inhibitor, and the results were statistically significant compared to control at 62.5 µM (85.2 ± 7.0%, *p* = 0.009), 125 µM (77.1 ± 5.9%, *p* < 0.001), 250 µM (70.7 ± 6.2%, *p* < 0.001) and 500 µM (60.4 ± 10.2%, *p* < 0.001) (Figure 2).

**Figure 3:**
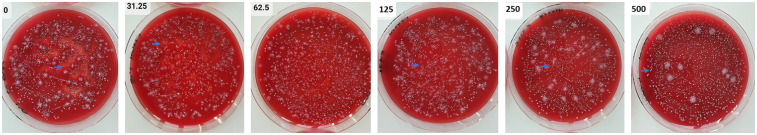
Agar plates with samples from 24 h 12-species biofilms were tested both without and with varying concentrations of S-0636. After performing a dilution series, aliquots of the biofilms were spread on agar plates and incubated anaerobically for 8 days. *P. gingivalis* (→) forms black colonies without the inhibitor; with increasing concentrations of the inhibitor, its colonies gradually lose their pigmentation.

It is of interest to note that *P. gingivalis* colonies lost pigmentation after high concentrations of the inhibitors (Figure 3).

### 3.3 mRNA expression of gingipains and FISH staining of biofilms

The analysis of mRNA expression of the gingipains after culturing the biofilm with 500 µM of the inhibitor resulted in nearly no difference for kgp (0.84 ± 0.17-fold) and a slight increase for rgpA (1.81 ± 0.47-fold) and rgpB (3.54 ± 0.17-fold) comparted to the untreated biofilm.

**Figure 4:**
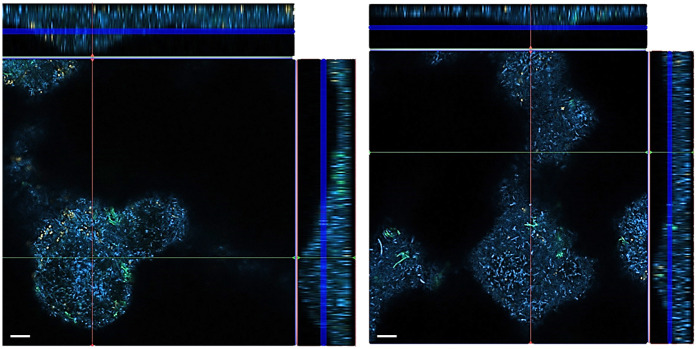
FISH images of 12-species biofilms cultured without (left) and with 500 µM of S-0636 (right). *P. gingivalis* appears yellow-orange, *T. forsythia* green, and all other bacteria are blue.

Confocal laser scanning microscopy (CLSM) photographs were taken from biofilms stained with three different FISH-probes. The images confirm the greater thickness of the control biofilms compared to the biofilm exposed to 500 µM of the [4,5-c]pyridine-based inhibitor (Figure 4).

**Figure 5:**
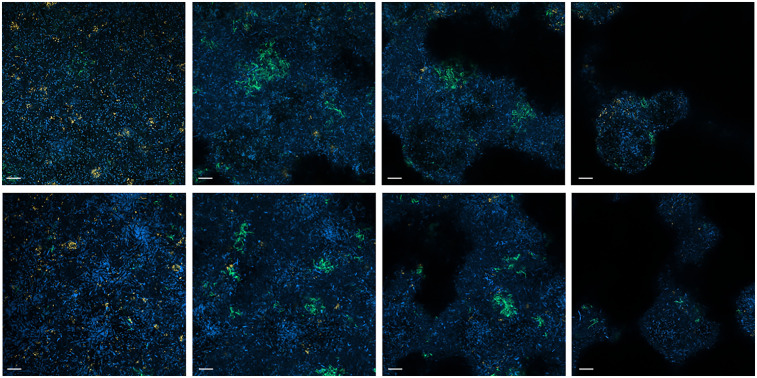
FISH images (630-fold magnification) of 12-species biofilms cultured without (upper row) and with 500 µM of S-0636 (lower row). Different layers of the biofilm from bottom to top (left to right) are visualized. *P. gingivalis* appears yellow-orange, *T. forsythia* green, and all other bacteria are blue. Bar 10 µm.

The photographs taken from different layers of the biofilm suggest that *P. gingivalis* is primarily located at the bottom, *T. forsythia* is found more in the middle layers of the biofilm, and it appears that *T. forsythia* forms clusters within the biofilms. Clear differences between the control biofilm and those cultured with 500 µM of the inhibitor are not visible (Figure 5).

### 3.4 Release of IL-1β and IL-10

**Figure 6:**
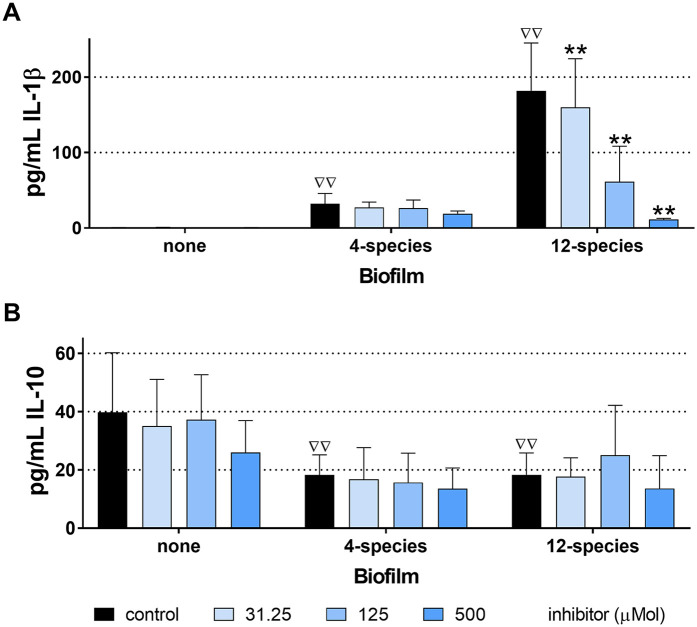
Levels of interleukin (IL)-1β **(A)** and IL-10 **(B)** released from MONO-MAC-6 cells after 4 h exposure to biofilms cultured without and with various concentrations of S-0636, *ΔΔp* < 0.01 compared to no biofilm (comparison only made for controls).**p < 0.01 vs. respective biofilm control.

Without bacterial stimulus, the MONO-MAC-6 cells did not release detectable IL-1β, but they did produce moderate amounts of IL-10. With the influence of biofilms, IL-1β levels increased and were highest in the case of the 12-species biofilm without inhibitor. The [4,5-c]pyridine-based inhibitor reduced the IL-1β release stimulated by the 12-species biofilm. In case of the 4-species biofilm, the IL-1β levels in the presence of the compound did not change statistically significantly compared to the 4-species biofilm without inhibitor (Figure 6A). Exposure to biofilms (both 4- and 12-species) decreased IL-10 levels. The inhibitor had no additional effect (Figure 6B). The MTT-test confirmed that S-0636 did not cause any cytotoxic reaction in the MONO-MAC-6 cells.

## 4 Discussion

The experiments focused on the effect of a [4,5-c]pyridine-based inhibitor on two defined biofilms. Two biofilms were included, a 4-species biofilm containing bacteria with a QC only *T. forsythia*, and a 12-species biofilm that also included *P. gingivalis* and *P. intermedia*.

One of the questions to be answered was whether there was a difference between the two biofilms. The 4-species biofilm was not affected by the inhibitor. Statistically significant differences were found only when using the 12-species biofilm. It might be linked to a stronger inhibition (lower K_i_-value) of *P. gingivalis* QC compared to *T. forsythia* QC (30).

Other questions were whether the inhibitor affects the biofilm as a whole and whether it alters the counts of specific bacteria. The total bacterial counts of the biofilm did not change. The counts of individual species, including *P. gingivalis*, *T. forsythia,* and the commensals (streptococci/actinomyces) remained stable. This is supported by the FISH images which clearly showed no differences in the counts of *P. gingivalis* and T. *forsythia*.

Only *P. intermedia* showed a concentration-dependent decrease in the number of cells in the biofilm treated with the inhibitor. A direct bactericidal activity of the inhibitor via QC can likely be ruled out, as the inhibitor's potency was higher compared to *P. gingivalis* QC (30). A possible reason for the reduction of *P. intermedia* numbers in the biofilm may be a decrease in the coaggregation ability between *P. gingivalis* and *P. intermedia.* An important coaggregation factor of *P. gingivalis* with *P. intermedia* is a protein from the outer membrane vesicles encoded by gingipain genes (31), whose secretion can be hindered.

The lower metabolic activity and biofilm biomass could indicate that impaired synergy in the biofilm formation. Since total bacterial counts were unaffected, the reduced biofilm biomass suggests an effect on the biofilm's matrix. Synergistic effects among periodontal bacteria have been described for *P. gingivalis.* Diffusible signaling molecules from *P. gingivalis* can interfere with *F. nucleatum's* metabolism and promote increased polysaccharide synthesis (32). A strong synergy in biofilm formation was observed for *P. gingivalis* and *T. denticola*, with gingipains playing a key role (33). RgpA appears to facilitate the growth of other oral species within a community, including *P. gingivalis*, *P. micra*, *F. nucleatum* and *Streptococcus constellatus* (34). Considering the specificity of the inhibitor, molecules being transported by the T9SS may also contribute to the formation of the biofilm matrix. According to functional categories, a high percentage of proteins involved in T9SS, outer membrane functions, and peptidoglycan biosynthesis carry an N-terminal Q (35). Pyroglutaminylation of the proteins by QC appears to stabilize them and protects them from degradation (35). It can be assumed that fewer proteins contributing to matrix formation are available; however, knowledge about proteins produced by *P. gingivalis*, *T. forsythia* and *P. intermedia* that contribute to biofilm matrix synthesis is remains limited.

The Arg-gingipain specific amidolytic activity, which correlates with *P. gingivalis* virulence, was clearly reduced by the QC inhibitor in a concentration-dependent manner. Our data suggest limited synthesis or processing of the gingipains. However, limited synthesis can be ruled out at least at the mRNA level. Synthesized prepro-gingipains undergo extensive post-translational proteolytic processing, similar to many other virulence factors, as they are first transported by the Sec system to periplasm and then through the T9SS (36). Blocking specific components of T9SS caused a loss of cell-associated gingipain activity (Kgp or total) and colony pigmentation (37, 38). In our study, we also observed a loss of colony pigmentation in *P. gingivalis* was observed. It has been shown that glutamine cyclization to pyro-glutamate plays a role in cell-associated activity of RgpA (15). The underlying mechanisms might be explained by the fact that proteins with N-terminal Q involved in T9SS (35) are either not functional or only partially functional when a QC inhibitor is present.

Furthermore, we evaluated the potential interaction between the QC inhibitor's effect on biofilm and the host response. For this, the pre-cultivated biofilm, with and without the inhibitor, was exposed to monocytic cells for 4 h, after which the release of interleukin (IL)-1β, a pro-inflammatory cytokine and IL-10, an anti-inflammatory cytokine was measured. In periodontitis, macrophages can be polarized into M1 or M2 types. M1-macrophages, which promote inflammation are characterized by high levels of pro-inflammatory cytokines, such as IL-1. In contrast, M2-macrophages, which facilitate anti-inflammatory functions, are marked by high levels of IL-10 (39). The initiation and progression of periodontal disease are driven by a dysregulated inflammatory response to a dysbiotic biofilm (40). This is supported by the *in vitro*-result showing high levels of IL-1β and sustained levels of IL-10 after exposure of the 12-species biofilm to monocytic cells. IL-1β is known to limit bacterial dissemination but mainly mediates periodontal inflammation, activation of osteoclasts and tissue destruction (41). A high level of IL-1β in saliva is an indicator for an occurring inflammation in periodontal tissue (42). Clinically, high IL-1β-level decrease after periodontal therapy, coinciding with improvements in periodontal clinical parameters (43). In the present study, IL-1β levels also decreased following application of the inhibitor.

Results in the 12-species biofilm were clearly dependent on the concentration of the inhibitor used. Since the compound will be incorporated into an oral health-care product for local application, it should be available at a sufficiently high concentration. It should be noted that in this *in vitro* study, the concentration of the inhibitor remained stable within the experimental setup. *In vivo*, depending on the application site, the flow of saliva or gingival crevicular fluid must be considered. The mean unstimulated salivary flow rate was measured at 287 µL/min (44), while stimulated salivary flow can reach up to 640 µL/min (45). Gingival crevicular fluid flow was approximately 0.5 µL/min (46). To address the issue of immediate dilution of the compound, it may be necessary to incorporate it into a slow-release device that can maintain the active concentration over an extended period (47).

The advantage of the study was using a clearly defined study design that allowed comparison of the two biofilms and different inhibitor concentrations. The studied variables enabled conclusions about the anti-biofilm, anti-virulence, and immunomodulatory properties of the inhibitor. However, the models only reflected part of the situation in the oral cavity; the biofilm consisted of up to 12 species, the interaction with host cells included only a monocytic cell line, and salivary or gingival crevicular fluid flow was not considered. These could be limitations of the study, highlighting the need for further *in vitro* research with more complex models. Finally, the efficacy of the inhibitor must be demonstrated through randomized clinical trials.

Overall, the compound might have potential for inclusion in oral health care products. It may alter the virulence of a dysbiotic biofilm, leading to a balanced pro- and anti-inflammatory responses. Since the inhibitor clearly acts in a concentration-dependent manner, it should be included at a sufficiently high concentration in an oral health care product.

## Data Availability

The raw data supporting the conclusions of this article will be made available by the authors, without undue reservation.
